# Analysis of Monacolins and Berberine in Food Supplements for Lipid Control: An Overview of Products Sold on the Italian Market

**DOI:** 10.3390/molecules26082222

**Published:** 2021-04-12

**Authors:** Sara Marcheluzzo, Marta Faggian, Mirella Zancato, Gregorio Peron

**Affiliations:** 1 Department of Pharmaceutical and Pharmacological Sciences, University of Padova, Via Marzolo 5, 35100 Padova, Italy; sara.marcheluzzo@studenti.unipd.it (S.M.); mirella.zancato@unipd.it (M.Z.); 2 Unired srl, Via Niccolò Tommaseo 69, 35131 Padova, Italy; nutraceutica@unired.it

**Keywords:** red yeast rice, monacolins, berberine, dietary supplements, nutraceuticals, mass spectrometry

## Abstract

The use of dietary supplements for the prevention and management of diseases associated with excess of lipids is spreading in Western countries. Supplements containing red yeast rice (RYR) and extracts from *Berberis* species, characterized, respectively, by the active compounds monacolin K (MK) and berberine (BBR), are sold in pharmacies as over the counter medicines (OTC) and in regular markets without the need of medical prescription and medical surveillance. However, MK is chemically identical to lovastatin, a drug commonly used to treat hypercholesterolemia, and is characterized by the same mechanism of action, pharmacokinetic profile and toxicity. On the other hand, although BBR-containing supplements are considered to be well-tolerated and safe, they frequently show poor standardization of active ingredients, and this could lead to lack of effects. In this work, with the aim to give an overview on the potency of RYR- and BBR-containing supplements available on the Italian market, we analyzed a pool of supplements bought from both local pharmacies and markets. Results confirm the data already published by other authors, showing scarce standardization of bioactives and discrepancy between the doses of bioactives reported by the manufacturers and the amounts resulting from analysis of the same products. Overall, our data represent a further proof that a strict legislation regulating the production and marketing of dietary supplements and a close monitoring of these products by food and drug regulatory organs is mandatory.

## 1. Introduction

Nowadays, pathologies related to an unhealthy lifestyle are widely diffused. Reduced physical activity and unhealthy dietary habits are two of the of the main causes of ailments such as obesity, hypertension and cardiovascular diseases. Many heart and circulatory dysfunctions are caused by the presence of a high percentage of circulating “bad” cholesterol, in general LDL [1]. Various therapeutic approaches have been approved for the treatment of pathologies caused by an excess of cholesterol depending on the severity of the pathology itself. In any case, prevention remains essential to improve health, which means paying attention to nutrition and lifestyle [2]. As a preventive strategy, patients may be advised to use dietary supplements “besides the diet but before the drugs” [3].

**Figure 1:**
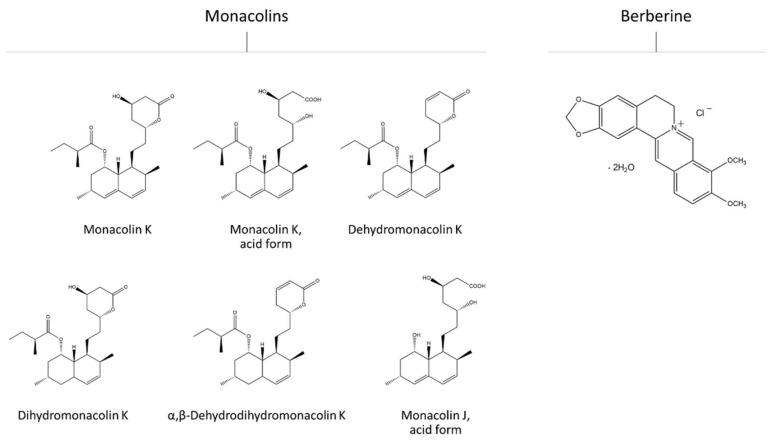
Chemical structures of the compounds evaluated in this study. Monacolins are the main bioactive constituents of dietary supplements containing red yeast rice, while berberine (BBR) is the main active compound of dietary supplements containing *B. aristata* powders and dried extracts. BBR is reported here as BBR chloride dihydrate, the same salt that was used as reference standard for BBR analysis in HPLC-DAD.

Since hypercholesterolemia is a crucial issue among the Western population, dietary supplements for the treatment of this dysfunction are widely studied and researched. Several products of natural origin have been reported as effective in lowering plasma cholesterol by acting with different mechanisms of action, and in recent years they have been frequently used as the main active ingredients of supplements available in pharmacies as over the counter medicines (OTC) or in regular markets without medical prescription. Among these, the most diffused on the market are the red yeast rice (RYR), characterized by the cholesterol-lowering compound monacolin K (MK), and berberine (BBR), an isoquinolone alkaloid isolated from the root, rhizome or bark of the Chinese herb *Coptis chinesis* and roots and stems of plants of the genus *Berberis* [4], together with plant phytosterols and policosanols. These extracts/compounds are often used as the sole active ingredients of dietary supplements or are combined with each other in products also containing probiotics, coenzyme Q and vitamins [5]. MK is a terpenoid whose chemical structure is identical to that of the drug lovastatin (Figure 1). Under acidic conditions, it adopts one of two forms, acidic or lactone, but only the acidic form plays a role in reducing blood lipid concentration [6]. Such as lovastatin, the mechanism of action behind the lipid-lowering activity of MK involves the competitive inhibition of the enzyme HMG-CoA reductase in the cholesterol synthesis pathway [6]. Additionally, the side effects associated with MK are similar to those reported for lovastatin, including muscle weakness and pain, hepatitis, and gastrointestinal symptoms such as nausea, vomiting, and diarrhea [7]. BBR (Figure 1) is a quaternary isoquinolinium salt that belongs to the class of protoberberine alkaloids. It has many chemical forms, such as hydrochloride, sulfate, citrate and phosphate salts [8]. The mechanism of action behind its cholesterol-lowering activity involves the upregulation of hepatic LDL receptor expression through a post-transcriptional mechanism that stabilizes the LDL-receptor mRNA [9]. Recent clinical trials show the efficacy of BBR and BBR-containing products in reducing total cholesterol [4], and they show scarce incidence of adverse effects, comprising diarrhea, constipation, and abdominal distension [8].

The growing expansion of dietary supplements (also referred to as nutraceuticals) on the market, their easy availability and the lack of specific legislation regulating their production and medical prescription have raised many questions about the safety and effectiveness of these products. In fact, first of all nutraceuticals do not yet have a recognized univocal definition that correctly classifies these products. According to the definition given by Stephen L. DeFelice in 1989, nutraceuticals are “food or part of a food, such as a food supplements, which have a medical or health benefit, including the prevention and treatment of illnesses” [10]. Hence, as there is no natural distinction between these products, at legislative and nonlegislative levels they are recognized as food supplements, which by definition have “non-medicinal” characteristics. The food supplement legislation, however, is much more limited than that for medicine, and this leads to facilitations for the production and the marketing of nutraceuticals [11].

Another aspect to consider regards the chemical composition of dietary supplements. In fact, bioactive natural compounds or extracts are often the main active ingredients of these products, although their biological effects and complex chemical compositions are not always completely known [12]. Furthermore, natural extracts often lack standardization of the main bioactive compounds, and this leads to a large variability of their amounts per serving, which could cause a lack of efficacy of the dietary supplements or, even worse, to unpredictable side effects [12]. Regarding nutraceuticals with lipid-lowering activity, articles published in the last 10 years have confirmed the heterogeneity of the products available on the market and the lack of standardization of the bioactive ingredients, showing frequent discrepancies between the amounts of bioactives reported by the manufacturers and those detected by the analysis of the same products [13,14,15,16,17].

Since, to the best of our knowledge, analyses of the content of monacolins and BBR in marketed dietary supplements for lipid control have been conducted only for products available in the U.S. [14,16,18], China [13,15], and France [17], in this work we aimed at recollecting data about dietary supplements available on the Italian market by determining the content of MK in RYR-containing dietary supplements, as well as the content of BBR in products containing this alkaloid as main lipid-lowering agent, both as a pure compound or as constituent of natural extracts. Considering the previously published reports on RYR and BBR supplements showing high variability in the amounts of MK and BBR among different product brands, we expected to observe a dose heterogeneity also in products available on the Italian market. Furthermore, considering that almost all previously published works on RYR supplements have focused mainly on the analysis of MK and did not consider other potential bioactive monacolins, in this work we also determined the variability of monacolin K, acid form (MKA), monacolin J, acid form (MJA), dihydromonacolin K, dehydromonacolin K, and α,β-dehydrodihydromonacolin K in different supplement brands sold in Italy, in order to give more exhaustive information about their potency and to point out potential safety concerns.

## 2. Results

### 2.1. Analysis of Food Supplements Containing Red Yeast Rice (Monacolin K and Other Monacolins)

**Figure 2:**
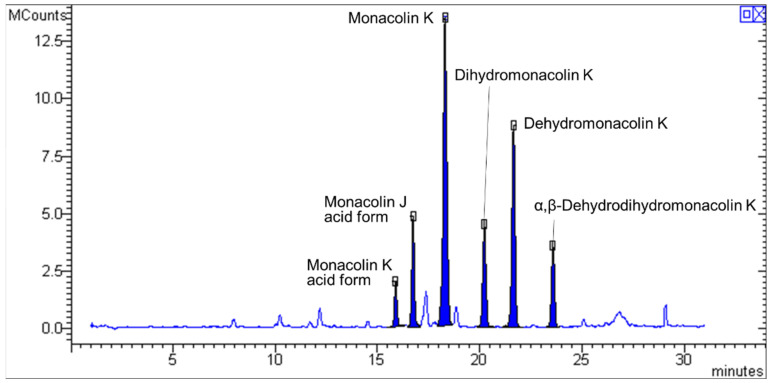
Representative chromatogram obtained from the analysis of a dietary supplement containing red yeast rice. The monacolins indicated in the figure were detected as [M+Na]^+^ adducts (*m/z* values for monacolin K acid form, monacolin J acid form, monacolin K, dihydromonacolin K, dehydromonacolin K and α,β-dehydrodihydromonacolin K = 445, 361, 427, 429, 409 and 411, respectively).

Ten different brands of commercially available supplements containing RYR as main lipid-lowering agent were evaluated for their content in MK and other potentially bioactive monacolins—namely, MKA MJA, dihydromonacolin K, dehydromonacolin K, and α,β-dehydrodihydromonacolin K [19]. Chemical structures of each monacolin are reported in Figure 1 and a representative chromatogram showing their corresponding peaks is reported in Figure 2.

Although clinical data about the efficacy of low-to-medium doses of MK in lowering circulating cholesterol are still controversial, daily doses of 2.5–10 mg are considered safe and well-tolerated, and a recent clinical trial reported the effectiveness of a daily 10 mg MK dose in improving lipid pattern [7]. In the supplements considered in our study, we observed a wide range of MK contents—i.e., from 1.51 to 9.88 mg/serving. Products 1, 2 and 3 were the ones with lower MK contents, although their compositions were characterized by the association of RYR with other ingredients with lipid-lowering activity (Table 1). Products 4 and 5 were also characterized by low MK dosages, although here the RYR was not associated with other bioactive compounds. Furthermore, a discrepancy between the MK amounts/serving reported by the manufacturer and the values that were obtained by the HPLC-MS analysis of products 4 and 5 was detected, and specifically, the analytical data showed significantly lower amounts than those reported by the manufacturer (Table 1). The same discrepancy was also observed in other products (e.g., products 1, 6 and 9), and, in general, the variability between the amounts/serving reported by the manufacturer and those observed after HPLC-MS analysis was between −49.7% and 37.7%, with products 1 and 5 showing the highest variability (Table 1).

Finally, considering other monacolins than MK, a high variability among the studied products was observed (Table 2). MJA, dihydromonacolin K, and dehydromonacolin K were the most abundant in the products with the highest MK contents (products 7–10), together with MKA in products at lower MK contents (products 1–5).

### 2.2. Analysis of Food Supplements Containing Berberine

Eight supplements containing BBR as main lipid-lowering agent were considered in this study, which are among those available on the Italian market. The chemical structure of BBR is reported in Figure 1. Results of the HPLC-DAD analyses conducted on these products are reported in Table 3. The source of BBR in all the studied products was *Berberis aristata*, either as dried bark/roots powder or extracts. As observed for RYR-containing supplements, a high variability in BBR content among the different product brands was detected. Results from clinical trials show the safety and the efficacy of BBR in reducing plasma lipids at daily doses of between 500 and 1500 mg, either as single compound and as part of standardized natural extracts, or in association with other bioactive compounds [20,21,22,23]. In the products considered in this study, the amount of BBR was between 97 and 667 mg/serving, although following the manufacturer’s recommendation regarding daily doses the variability range was increased (Table 3). Furthermore, for BBR-containing products we also observed a discrepancy between the BBR amounts/serving reported by the manufacturer and those detected by HPLC-DAD analysis, although, differently from what was observed for RYR-containing products, the amounts resulting from HPLC-DAD analysis were higher than those declared on the brand (Table 3), with variability between 0.2% for product 12 and 59.7% for product 14.

## 3. Discussion

The use of dietary supplements for health-promoting purposes and for the prevention and treatment of mild diseases is becoming a spread practice worldwide. In Europe, these products lack a restrictive legislation concerning their production and marketing [11], and this is contributing enormously to their rapid expansion and is attracting an increasing number of consumers who can buy dietary supplements from pharmacies and markets without the need of medical prescriptions and bypassing medical surveillance. Considering the Italian scenario, about 32 million citizens use dietary supplements, and among these, 18 million use them on a regular (daily or weekly) basis. It has been estimated that Italy’s nutraceutical market grew by 126% in the period between 2008 and 2018, during which revenues increased from EUR 1.3 billion to EUR 3.3 billion [24]. Dietary supplements for lipid control based on RYR represented the fourth most important class of nutraceuticals in terms of values during 2017–2018 after probiotics, mineral supplements, and multivitamin–multimineral supplements [24].

Despite dietary supplements being often distributed without proper medical surveillance, they contain bioactive ingredients that could exert either positive or negative effects on health depending on their use [25], and the literature regarding their efficacy and safety is often contradictory. Furthermore, the lack of a strict control by food and drug regulatory organs can lead to the marketing of products that, especially in the case of herbal nutraceuticals, might have been scarcely characterized in terms of their contents of potential bioactive (and potentially harmful) components [26]. As already highlighted by other authors, these issues frequently occur for food supplements with lipid-reducing properties, such as those containing RYR and BBR as the main active ingredients [17,18]. Commercial RYR powders and extracts often present scarce standardization of MK and other potentially bioactive monacolins, whose bioactivity and safety have not been well-established yet [27]. This is associated with a wide variability of monacolin contents among different supplement brands, which can cause variations of efficacy and toxicity of such products. Most importantly, MK is characterized by the same chemical structure of lovastatin, and hence it shows equivalent mechanism of action and similar side effects [28]. An investigation conducted within the Italian Surveillance System of Natural Health Products showed that the safety profile of RYR reflects that of statins, reporting frequent adverse reactions related to RYR products such as myalgia and/or increase in creatine phosphokinase, liver injury, gastrointestinal reactions and cutaneous reactions [28]. Daily MK doses of 2.5–10 mg are considered safe and well-tolerated, and a recent clinical trial reported the effectiveness of a daily 10 mg MK dose in improving lipid pattern [7]. Additionally, the pharmacokinetic profile of MK resembles that of lovastatin, and both lovastatin and MK from RYR have been reported to be inhibitors of cytochrome P450 enzymes (CYP3A4, CYP1A2 and CYP2C19) and P-glycoprotein [29]. These activities can lead to supplement–drug interactions, especially with cyclosporin, itraconazole, clarithromycin, verapamil, aprepitant, amiodarone, ritonavir and other cholesterol-lowering agents such as fibrates [30].

In the RYR-containing supplements included in our study, we observed a marked variability of MK content, i.e., from 1.51 to 9.88 mg/serving. Products 1, 2 and 3 were those with lower MK contents, but their compositions were characterized by the association of RYR with other ingredients with hypocholesterolemic activity such as coenzyme Q10, policosanols, *Ipomea batatas*, *Olea europea* and *Citrus* peels dried extracts, which justifies the low MK amount. In fact, clinical trials showed the efficacy of nutraceuticals containing low MK doses (i.e., 3 and 5 mg) in association with BBR (500 mg) and plant phytosterols (800 mg), respectively, in improving plasma LDL cholesterol, HDL cholesterol and total glycerides [31,32,33]. Nevertheless, none of the products included in our study showed an MK amount/serving that could be considered as potentially toxic (i.e., >10 mg), also considering the consumption of servings/day suggested by the manufacturers. However, a high inter-brand variability and inhomogeneity of the profile of other potentially bioactive monacolins was observed, and this is an important aspect that should be carefully taken into consideration, especially by manufacturers and clinicians. Our results are partially in line with those already published by other authors regarding RYR nutraceuticals sold in the U.S. [14,16], China [13,15], and France [17], reporting MK doses between 0.10 and 11 mg/serving, approximately, and lack of standardization of other monacolins such as MKA, monacolin J, dehydromonacolin K and dihydromonacolin K, whose amounts are usually not reported by manufacturers.

Similar results were obtained for the BBR-containing supplements analyzed in this study. As for RYR supplements, a large variability in the amounts of BBR/serving was observed among the different brands, as well as frequent discrepancies between the amounts detected by HPLC-DAD and those declared by manufacturers. Additionally, in this case, our results regarding nutraceuticals sold in Italy are in line with previously published data about BBR products from the U.S. [18]. However, differently from RYR supplements, in all the BBR products where variations between quantitative analytical data and amounts reported by manufacturers were observed, the detected amounts were higher, and variability was between 0.2% and 59.7%. Nevertheless, daily BBR doses between 500 mg and 1500 mg are considered well-tolerated and safe [20,21,22,23]. Furthermore, results from a recently published dose–response meta-analysis highlight greater improvements of obesity indices at BBR dosages of >1000 mg/day [34]. Following the manufacturer’s recommendations regarding the servings/day, daily BBR doses for the products included in our study would not exceed the amounts considered as safe and well-tolerated. On the other hand, several of these products lack efficacy in improving lipid levels, considering that in many cases the daily doses of BBR that consumers should consume following the same recommendations would be <1000 mg/day. These results show, again, the need for regulatory organs, manufacturers and clinicians to act together to establish a more restricted regulation regarding the production and the marketing of BBR-containing supplements, as well as the need for improvements in the clinical surveillance on their use.

## 4. Materials and Methods

### 4.1. Materials

Methanol (purity ≥ 99.9%) was used for sample preparation and was purchased from Sigma-Aldrich (Milan, Italy). Formic acid (purity ≥ 95%) and acetonitrile (purity ≥ 99.9%) were used to prepare the mobile phases for HPLC analyses and were purchased from Sigma-Aldrich (Milan, Italy), while water was Milli-Q grade (18.2 MΩ.cm at 25 °C) and was obtained through a Millipore filtration system (Billerica, MA, US). MK and BBR chloride dihydrate standards were purchased from Sigma-Aldrich (Milan, Italy).

### 4.2. Supplements and Sample Preparation

In this study, food supplements containing RYR or BBR were considered that are to be among the most popular nonmedicinal products with lipid-lowering activity [35]. Ten different supplement brands containing RYR and eight containing BBR were bought during September–October 2020 from pharmacies and markets in the North-Eastern region of Italy and, to be included in the study, they had to contain RYR (as dried powder or extract) or BBR (pure compound or as dried *B. aristata* powder or extract) as main active ingredients. All the products considered in this study are currently produced by Italian manufacturers (names are not reported to avoid conflicts of interest) and are regularly sold without the need of medical prescription. Supplements produced by local pharmacies were not included in the study.

For sample preparation, tablets or the content of capsules were weighted and grinded using a mortar and pestle, and the obtained powder was extracted at RT using methanol in ultrasound bath for 20 min. After centrifuging the samples at 13,000 rpm for 10 min, the supernatants (2 mL) were filtered through 0.45 μm PTFE membrane filters and then transferred to amber-glass vials for HPLC analysis.

### 4.3. HPLC-MS Analysis of Monacolins in RYR-Containing Supplements

The contents of MK, MKA, MJA, dihydromonacolin K, dehydromonacolin K, and α,β-dehydrodihydromonacolin K in dietary supplements were analyzed using an HPLC-MS approach. Chromatographic separation was conducted on an Agilent 1260 binary pump equipped with an Agilent Extend C18 column (4.6 × 150 mm, 3.5 µm) as the stationary phase and coupled to a Varian 320 (Triple Quadrupole) mass spectrometer (MS) operating in positive ion mode (ESI+). A mixture of 0.1% formic acid in water (A) and acetonitrile (B) was used as the mobile phase, and the flux was 1 mL/min. The linear gradient was as follows: 0 min, 10% B; 30 min, 95% B; 32 min, 10% B. The column was then left to re-equilibrate until 35 min. The [M+Na]^+^ adducts of the six monacolins were monitored as they yielded a higher signal compared to the corresponding [M+H]^+^ ions. Monitored *m/z* values are reported in Table 4 [15]. Quantification of MK and semiquantification of the other monacolins was performed using a linear calibration curve obtained by analyzing standard MK solutions in the concentration range 2–20 µg/mL. The equation of the resulting curve was *y* = 984867*x*. All the samples were analyzed in triplicate, and the results were reported as means ± standard deviation (SD). Limit of detection (LOD), limit of quantitation (LOQ), intraday and interday precision were estimated using the FDA validation protocol for analytical methods [36]. For MK, LOD = 0.1 µg/mL and LOQ = 0.6 µg/mL, while relative standard deviations for intraday and interday precision were 0.1−7.1% and 1.2−12.6%, respectively.

### 4.4. HPLC-DAD Analysis of BBR in Supplements Containing B. aristata

BBR was analyzed using an HPLC-DAD approach. Chromatographic separation was conducted on an Agilent 1260 binary pump equipped with an Agilent ZORBAX Eclipse XDB-C8 (4.6 × 150 mm, 5 µm) as the stationary phase and coupled to an Agilent 1260 diode array detector (DAD) operating at λ = 350 nm (λ of BBR maximum absorbance). A mixture of 1% formic acid in water (A) and acetonitrile (B) was used as mobile phase, and the flux was 1 mL/min. The linear gradient was as follows: 0 min, 10% B; 11 min, 100% B; 12 min, 100% B; 12.1 min, 10% B. The column was then left to re-equilibrate for 14 min. Quantification of BBR was performed using a linear calibration curve obtained by analyzing standard BBR solutions in the concentration range 10–100 µg/mL. The equation of the resulting curve was *y* = 2 × 10^7^*x.* All the samples were analyzed in triplicate, and the results were reported as means ± SD. Method validation parameters were estimated as previously described. LOD = 0.15 µg/mL and LOQ = 0.7 µg/mL, while relative standard deviations for intraday and interday precision were 0.4−8.3% and 2.1−15.1%, respectively.

## 5. Conclusions

In this work, we aimed at monitoring the contents of monacolins and BBR in a representative sample of RYR- and BBR-containing nutraceuticals available on the Italian market in order to give an overview of their potency. A large amount of variability in the doses of MK and other monacolins and BBR was detected among the different product brands and also between the amounts of active compounds declared by the manufacturers and those resulting from HPLC analysis. Our work represents the first report on the potency and variability of RYR and BBR supplements currently sold in Italy. The results here presented reflect the data previously published by other authors regarding similar products available in other European and extra-European countries, and they highlight the need of a more restricted control by Italian and European food and medicine regulatory organs of the production, standardization and marketing of nutraceuticals for lipid control. Moreover, the results reported here show the lack of standardization and the high variability in the content of other potentially bioactive monacolins other than MK in commercial nutraceuticals—namely, MKA, MJA, dihydromonacolin K, dehydromonacolin K and α,β-dehydrodihydromonacolin K. Overall, we emphasize the necessity for the manufacturers to provide information about the content of these monacolins and for further research to be conducted to assess their pharmacological properties and adverse effects in order to determine their safety and their contribution to the whole activity of RYR.

## Figures and Tables

**Table 1:** Amounts of active ingredients, pharmaceutical forms and labelled information of food supplements containing red yeast rice as the main active ingredient. Detected MK amounts are reported as mean mg/serving ± SD.

N.	Labelled Active Ingredient	Formulation	Recommended Servings/Day *	MK/Serving (mg) *	Detected MK/Serving (mg)	Other Active Ingredients (mg/Serving) **
1	RYR powder (100 mg, 3% MK)	Tablets	1	3	4.13 ± 0.07	*Ipomea batatas* d.e. (160 mg); *Citrus reticulata* peels d.e. (40 mg); *Olea europea* fruit d.e. (25 mg); Coenzyme Q10 (5 mg)
2	RYR powder (100 mg, 3% MK)	Tablets	1	3	3.07 ± 0.03	*Garcinia cambogia* d.e. (50 mg); *Citrus bergamia* peels d.e. (50 mg); Coenzyme Q10 (20 mg)
3	RYR powder (100 mg, 3% MK)	Tablets	1	3	2.58 ± 0.04	Coenzyme Q10 (100 mg); Policosanols (20 mg); astaxanthin (2 mg)
4	RYR powder (200 mg, 1.5% MK)	Tablets	1	3	2.50 ± 0.02	ND
5	RYR d.e. (262.5 mg, 0.4% MK; 130 mg, 1.5% MK)	Capsules	1	3	1.51 ± 0.09	ND
6	RYR powder (167 mg, 3% MK)	Capsules	2	5	2.91 ± 0.11	ND
7	RYR d.e. (200 mg, 5% MK)	Tablets	1	10	9.19 ± 0.05	ND
8	RYR d.e. (200 mg, 5% MK)	Tablets	1	10	9.88 ± 0.05	ND
9	RYR d.e. (200 mg, 5% MK)	Tablets	1	10	8.99 ± 0.01	ND
10	RYR powder (200 mg, 5% MK)	Tablets	1	10	9.17 ± 0.22	ND

* As reported by manufacturers. ** The amounts of active ingredients are those reported by manufacturers. RYR: red yeast rice; MK: monacolin K; d.e.: dried extract.

**Table 2:** Monacolin K, acid form, monacolin J, acid form, dihydromonacolin K, dehydromonacolin K and α,β-dehydrodihydromonacolin K contents in dietary supplements containing red yeast rice as the main active ingredient. Values are reported as mean mg/serving ± SD.

N.	MKA	MJA	Dihydromonacolin K	Dehydromonacolin K	α,β-Dehydrodihydromonacolin K
1	0.84 ± 0.01	0.76 ± 0.01	0.54 ± 0.01	0.63 ± 0.04	0.25 ± 0.02
2	0.41 ± 0.01	0.35 ± 0.01	0.60 ± 0.01	0.32 ± 0.01	0.12 ± 0.00
3	0.54 ± 0.02	0.68 ± 0.01	0.65 ± 0.01	0.50 ± 0.02	0.18 ± 0.01
4	0.22 ± 0.03	0.18 ± 0.02	0.24 ± 0.01	<0.1	<0.1
5	0.70 ± 0.01	0.65 ± 0.04	0.60 ± 0.01	1.36 ± 0.08	0.52 ± 0.02
6	0.60 ± 0.05	0.46 ± 0.01	1.47 ± 0.12	1.30 ± 0.05	0.55 ± 0.01
7	2.01 ± 0.12	2.71 ± 0.10	2.90 ± 0.10	5.02 ± 0.21	1.88 ± 0.10
8	1.98 ± 0.12	2.96 ± 0.09	2.21 ± 0.10	4.02 ± 0.04	1.67 ± 0.03
9	1.06 ± 0.04	2.55 ± 0.10	2.86 ± 0.13	4.31 ± 0.10	2.00 ± 0.08
10	1.25 ± 0.10	1.55 ± 0.08	1.13 ± 0.02	2.21 ± 0.10	0.78 ± 0.02

MKA: Monacolin K, acid form; MKJ: monacolin J, acid form.

**Table 3:** Amounts of active ingredients, pharmaceutical forms and labelled information of food supplements containing berberine as the main active ingredient. Detected berberine amounts are reported as mean mg/serving ± SD.

N.	Labelled Active Ingredient	Formulation	Recommended Servings/Day *	BBR/Serving (mg) *	Detected BBR/Serving (mg)	Other Active Ingredients (mg/Serving) **
11	*B. aristata* DC bark d.e. (100 mg, 97% BBR)	Tablets	2	97	97.41 ± 0.23	Coenzyme Q10 (10 mg); Olive leaves dry extract (50 mg, 6% oleuropein); olive fruit dry extract (50 mg, 10% hydroxytirosol), *Silybum marianum* dry extract (40 mg, 2% silimarin)
12	*B. aristata* DC bark d.e. (100 mg, 97% BBR)	Tablets	2	97	97.20 ± 0.08	ND
13	*B. aristata* DC bark d.e. (250 mg, 97% BBR)	Tablets	1 or 2	242.5	255.01 ± 34.20	beta (1,3)-Glucan (100 mg); “Cinnamon” dry extract (100 mg, 1.6% MHCP)
14	*B. aristata* DC bark and roots d.e. (294 mg, 85% BBR)	Capsules	2	250	398.43 ± 29.22	ND
15	*B. aristata* DC d.e. (294 mg, 85% BBR)	Tablets	2	250	280.33 ± 21.10	ND
16	*B. aristata* DC bark d.e. (450 mg, 83.6% BBR)	Tablets	2	376.2	514.66 ± 11.21	ND
17	*B. aristata* DC d.e. (BBR standardization unknown)	Tablets	1	500	663.17 ± 33.01	Monacolin K (10 mg; from red yeast rice powder)
18	*B. aristata* DC bark d.e. (BBR standardization unknown)	Tablets	2	500	561.12 ± 21.50	ND

*As reported by manufacturers. ** The amounts of active ingredients are those reported by manufacturers. BBR: berberine; d.e.: dried extract.

**Table 4:** Information about the molecular formula, nominal mass, adduct types and monitored *m/z* values for monacolins considered in this study.

Compound	Molecular Formula	Nominal Mass (Da)	Adduct Type	*m/z*
Monacolin K	C_24_H_36_O_5_	404	[M + Na]^+^	427
Monacolin K, acid form	C_24_H_38_O_6_	422	[M + Na]^+^	445
Monacolin J, acid form	C_19_H_30_O_5_	338	[M + Na]^+^	361
Dihydromonacolin K	C_24_H_38_O_5_	406	[M + Na]^+^	429
Dehydromonacolin K	C_24_H_34_O_4_	386	[M + Na]^+^	409
α,β-Dehydrodihydromonacolin K	C_24_H_36_O_4_	388	[M + Na]^+^	411

## Data Availability

Not applicable.

## Sample Availability

All the samples considered in this work are available from the authors.
